# Retrospective chart review of melanoma outcomes in non‐Hispanic black patients and case‐matched non‐Hispanic white patients

**DOI:** 10.1002/ski2.204

**Published:** 2023-01-05

**Authors:** Nicole Edmonds, Braden Miller, Arturo Saavedra

**Affiliations:** ^1^ Department of Dermatology University of Virginia Charlottesville Virginia USA

To the Editor

1

It has long been studied that non‐Hispanic black (NHB) patients have a lower incidence rate of melanoma compared to non‐Hispanic white (NHW) patients owing to the photoprotective effect of melanin pigment in the skin.^1^ Though NHB patients have a lower risk of melanoma, they are typically diagnosed at a later stage and have poorer survival rates than NHW patients.^2^ Previous analyses have found that acral lentiginous melanoma (ALM), which typically presents on the palms and soles and is associated with poor survival outcomes, is more common in NHB patients and could be contributing to these racial disparities.^3^, ^4^ Other social determinants of health (SDOH) including insurance status, distance a patient lives from the hospital, and time from diagnosis to treatment could be contributing as well. To test this data at our institution, we performed a retrospective chart review of all NHB patients presenting to the University of Virginia with melanoma after 2010, when the electronic medical record was first implemented. A total of 24 NHB patients were diagnosed with melanoma within this 11‐year period. Twenty four NHW patients were case matched, controlling for the histological depth of invasion, which is the most important prognostic factor for cutaneous melanoma.^5^ Outcomes were compared across groups.

Depth was controlled within 0.5 mm in all cases with a median depth of 1.79 mm (range 0–10 mm) in the NHB patients and 1.8 mm (range 0–10 mm) in the NHW patients (*p* = 0.984). Though depth was controlled, NHW patients presented at slightly advanced stages due to more frequent nodal involvement (7 NHW, 3 NHB) at the time of presentation. Despite the slightly favourable initial stage, we found that the NHB patients had worse outcomes. Eight NHB patients developed metastatic disease and five patients passed away due to their melanoma, compared to five NHW patients with metastatic disease and three patients passing away due to their melanoma. Demographic variables such as age (NHB median 69 years [29–90 years], NHW median 69 years [31–90 years]) and gender (10/24 NHB males, 12/24 NHW males) were similar between groups. The location of and histologic subtype of melanoma did vary between groups. Sixteen NHB patients presented with melanoma on the hands or feet compared to 0 NHW patients (*p* < 0.001). In contrast, 11 NHW patients presented with melanoma on the head/neck while only 1 NHB patient presented with melanoma on the head/neck (*p* < 0.001). When comparing histologic subtype, 12 NHB patients presented with ALM while only 1 NHW patient presented with ALM (*p* < 0.001). Furthermore, 7 out of 8 NHB patients with progressive disease had ALM, though there was no statistically significant difference in tumour stage (*p* = 0.27) or thickness (*p* = 0.62) amongst NHB patients with ALM compared to other subtypes of melanoma. When analyzing SDOH, NHB patients were found to live closer to the hospital (NHB median 43.5 miles (1.3–122 miles), NHW median 69.0 miles (13–218 miles), (*p* = 0.0098) and have a shorter time from diagnosis to treatment (NHB median 23 days (7–69 days), NHW median 30 days (9–81 days), *p* = 0.55). There was no statistically significant difference in the distance patients lived from the hospital (*p* = 0.48) nor the time from diagnosis to surgery (*p* = 0.98) amongst patients with progressive disease compared to those with stable disease. We also studied the effect of insurance status on outcomes. Four NHB patients did not have insurance, compared to three NHW patients. Two of the four NHB patients without insurance had metastatic disease, one of which was due to refusal of therapy. All three of the NHW patients without insurance had stable outcomes.

It has previously been hypothesized that ALM confers a worse prognosis due to a later stage of disease or tumour thickness.^3^ However, the data from this small retrospective chart review revealed that the acral lentiginous subtype of melanoma was associated with a worse prognosis even when tumour thickness and tumour stage were controlled. Even when the more obvious SDOH are mitigated, the biologic behaviour of melanoma in NHB patients still appears to be more morbid, perhaps owing to the distinct sets of genetic alterations in melanoma.^6^ Other SDOH could be contributing as well such as socioeconomic status, education level, or access to transportation; however, these are not routinely screened at clinic appointments. We present this data to emphasize the need for coupling SDOH to future research in the biology of melanoma, as well as to emphasize the importance of better inquiry and documentation of SDOH, which play a key role in health outcomes. This data is summarized in Tables 1 and 2.

**TABLE 1:** Outcome data for non‐Hispanic black patients

Legal sex	Age	Stage	Tumour thickness (mm)	Initial treatment	Metastatic disease (Y/N)	Survival	Insurance coverage (Y/N)	Time from diagnosis to surgery (Days)	Distance from UVA (miles)
Male	76	Local	0	Pt declined therapy	Y	Alive	N	N/A	40
Female	90	Stage IA (T1aN0M0)	0.9	Pt declined therapy	Y	Deceased	N	N/A	6.8
Female	37	Stage IIA (T2bN0M0)	1.5	WLE + SLNB	Y	Alive	Y	26	48
Female	73	Stage IB (T2aN0M0)	1.56	WLE + SLNB	Y	Deceased	Y	33	75
Male	58	Stage IV (T2aN3M1)	2	Pembrolizumab	Y	Alive	Y	69	58
Female	84	Stage IIB (T3bNxM0)	3.05	Amputation (SLNB not performed due to multiple co‐morbidities)	Y	Deceased	Y	15	27
Female	84	Stage IIA (T3aN0M0)	3.32	WLE + SLNB	Y	Deceased	Y	7	87
Female	73	Stage IV (T4bNxM1)	6.5	Amputation (SLNB not performed because initial biopsy read as melanoma in situ)	Y	Deceased	Y	13	36
Female	76	Local	0	WLE	N	Alive	Y	44	42
Female	77	Local	0	WLE	N	Alive	Y	28	49
Female	78	Local	0	Amputation + SLNB	N	Alive	Y	15	46
Male	66	Local	0	Excisional biopsy (adequate margins, WLE not necessary)	N	Alive	Y	N/A	37
Female	46	Stage IA (T1bN0M0)	0.9	WLE	N	Alive	Y	63	25
Female	29	Stage IA (T1aN0M0)	1	WLE + SLNB	N	Alive	Y	10	122
Male	69	Stage IIA (T2bN0M0)	1.25	WLE + SLNB	N	Alive	N	12	117
Male	58	Stage IIA (T2bN0M0)	1.78	WLE + SLNB	N	Alive	N	20	91
Male	53	Stage IIA (T2bN0M0)	1.8	WLE + SLNB	N	Alive	Y	55	40
Female	81	Stage IIB (T3aNxM0)	2.5	WLE (pt declined SLNB)	N	Alive	Y	40	6.1
Female	61	Stage IIIB (T3aN1M0)	2.8	WLE + SLNB	N	Alive	Y	28	34
Male	76	Stage IIC (T4bN0M0)	4	WLE + SLNB	N	Alive	Y	7	1.3
Female	69	Stage IIA (T3aN0M0)	4.5	Amputation + SLNB	N	Alive	Y	36	92
Male	34	Stage IIIC (T4bN2M0)	5	WLE + SLNB (pt declined adjuvant therapy)	N	Alive	Y	23	45
Male	63	Stage IIC (T4bN0M0)	7	Amputation + SLNB	N	Alive	Y	10	46
Male	61	Stage IA (T1aN0M0)	10	Amputation + SLNB	N	Alive	Y	20	22

Abbreviations: SLNB, sentinel lymph node biopsy; WLE, wide local excision.

**TABLE 2:** Outcome data for non‐Hispanic white case matched control patients

Legal sex	Age	Stage	Tumour thickness (mm)	Initial treatment	Metastatic disease (Y/N)	Survival	Insurance coverage (Y/N)	Time from diagnosis to surgery (Days)	Distance from UVA (miles)
Male	76	Stage IIA (T3aN0M0)	3.8	WLE + SLNB (pt declined adjuvant immunotherapy)	Y	Alive	Y	49	40
Male	69	Stage IIB (T4aN0M0)	4.5	WLE + SLNB (pt declined adjuvant immunotherapy)	Y	Deceased	Y	9	67
Male	31	Stage IV (T4bN1M1)	6	IL‐2 + Ipilimumab + Vemurafenib + Pembrolizumab + Resection + RT	Y	Deceased	Y	30	68
Female	90	Stage IIIB (T4bN1M0)	7	WLE + SLNB (pt declined adjuvant immunotherapy)	Y	Deceased	Y	81	88
Male	66	Stage IIID (T4N3M0)	10	WLE + radical neck dissection + RT	Y	Alive	Y	21	75
Female	76	Local	0	Mohs	N	Alive	Y	14	68
Female	81	Local	0	WLE	N	Alive	Y	19	41
Male	58	Local	0	WLE	N	Alive	Y	32	13
Male	53	Local	0	Excisional biopsy + Imiquimod × 12 weeks (pt declined WLE)	N	Alive	Y	N/A	218
Male	61	Local	0	WLE	N	Alive	N	31	38
Male	34	Stage IA (T1aN0M0)	0.7	WLE	N	Alive	N	13	96
Male	57	Stage IB (T1bN0M0)	1	WLE + SLNB	N	Alive	Y	51	56
Female	37	Stage IB (T1bN0M0)	1	WLE + SLNB	N	Alive	Y	33	77
Male	58	Stage IB (T2aN0M0)	1.2	WLE + SLNB	N	Alive	Y	28	118
Female	64	Stage IB(T2aN0M0)	1.45	WLE + SLNB	N	Alive	Y	39	41
Female	38	Stage IIIA (T2aN1M0)	1.6	WLE + SLNB + adjuvant 6MHP	N	Alive	Y	42	67
Female	77	Stage IIB (T3bN0M0)	1.8	WLE + SLNB	N	Alive	Y	44	77
Female	84	Stage IB (T2aN0M0)	1.8	WLE + SLNB	N	Alive	N	10	57
Female	69	Stage IIIB (T3aN2M0)	2.15	WLE + SLNB + adjuvant Nivolumab	N	Alive	Y	34	177
Male	82	Stage IIA (T3aN0M0)	2.4	WLE + SLNB	N	Alive	Y	30	67
Female	84	Stage IIA (T3aN0M0)	2.85	WLE + SLNB	N	Alive	Y	29	83
Female	73	Stage IIIB (T3aN1M0)	3.1	WLE + SLNB (pt declined adjuvant immunotherapy)	N	Alive	Y	15	104
Male	72	Stage lllB (T3aN2M0)	3.65	WLE + SLNB (pt declined adjuvant immunotherapy)	N	Alive	Y	27	113
Female	78	Stage IIC (T4bN0M0)	5.01	WLE + SLNB (pt declined adjuvant immunotherapy)	N	Alive	Y	20	70

Abbreviations: MHP, melanoma helper peptide; RT, radiation therapy; SLNB, sentinel lymph node biopsy; WLE, wide local excision.

## CONFLICT OF INTEREST

None to declare.

## AUTHOR CONTRIBUTIONS

**Nicole Edmonds**: Conceptualization (Lead); Data curation (Lead); Formal analysis (Lead); Investigation (Lead); Methodology (Lead); Project administration (Lead); Visualization (Lead); Writing – original draft (Lead); Writing – review & editing (Lead). **Braden Miller**: Data curation (Equal); Investigation (Equal); Writing – review & editing (Supporting). **Arturo Saavedra**: Conceptualization (Equal); Project administration (Lead); Supervision (Lead); Writing – review & editing (Equal).

## FUNDING INFORMATION

This article received no specific grant from any funding agency in the public, commercial, or not‐for profit sectors.

## ETHICS STATEMENT

Reviewed and exempt by IRB; tracking # HSR210009.

## Data Availability

The data that support the findings of this study are available from the corresponding author upon reasonable request.
